# Maintain the light, long-term seasonal monitoring of luminous capabilities in the brittle star *Amphiura filiformis*

**DOI:** 10.1038/s41598-024-64010-x

**Published:** 2024-06-09

**Authors:** Constance Coubris, Laurent Duchatelet, Jérôme Delroisse, Wendy Shirley Bayaert, Laura Parise, Marie Christine Eloy, Christophe Pels, Jérôme Mallefet

**Affiliations:** 1 Marine Biology Laboratory, Earth and Life Institute, UCLouvain, Croix du Sud 3, 1348 Louvain-la-Neuve, Belgium; 2 Biology of Marine Organisms and Biomimetics Unit, Research Institute for Biosciences, UMONS, 23 Place du Parc, 7000 Mons, Belgium; 3 Institut des Sciences de la Vie, UCLouvain, Croix du Sud 4-5, 1348 Louvain-la-Neuve, Belgium; 4 Laboratory of Cellular and Molecular Biology, GIGA Institute, 4000 Liège, Belgium

**Keywords:** Ophiuroid, Luminescence, Dietary acquisition, Coelenterazine, Luciferase, Autofluorescence, Animal physiology, Marine biology

## Abstract

The European brittle star *Amphiura filiformis* emits blue light, via a *Renilla*-like luciferase, which depends on the dietary acquisition of coelenterazine. Questions remain regarding luciferin availability across seasons and the persistence of luminous capabilities after a single boost of coelenterazine. To date, no study has explored the seasonal, long-term monitoring of these luminous capabilities or the tracking of luciferase expression in photogenic tissues. Through multidisciplinary analysis, we demonstrate that luminous capabilities evolve according to the exogenous acquisition of coelenterazine throughout adult life. Moreover, no coelenterazine storage forms are detected within the arms tissues. Luciferase expression persists throughout the seasons, and coelenterazine's presence in the brittle star diet is the only limiting factor for the bioluminescent reaction. No seasonal variation is observed, involving a continuous presence of prey containing coelenterazine. The ultrastructure description provides a morphological context to investigate the green autofluorescence signal attributed to coelenterazine during luciferin acquisition. Finally, histological analyses support the hypothesis of a pigmented sheath leading light to the tip of the spine. These insights improve our understanding of the bioluminescence phenomenon in this burrowing brittle star.

## Introduction

Bioluminescence allows organisms to emit light through a biochemical reaction. Light emission results from the oxidation of a substrate called luciferin, catalyzed by an enzyme called luciferase^1^. This catalysis forms a transient intermediate, often a cyclic peroxide, that breaks down to produce oxyluciferin. The latter is electronically excited and returns to the ground state by emitting photons^1^. Through the years, the bioluminescent components have been extensively chemically studied^2–7^. To date, 9 natural luciferins have been chemically characterized along with dozens of associated luciferases^3,4^. In some cases, both components are grouped in a single unit protein called photoprotein, which requires a co-factor, often an ion, to trigger the light emission reaction^1,8^. Among luciferase and photoproteins, a recent study identified at least 12 homologous groups of bioluminescent proteins in the living kingdom^4^.

To date, five types of luciferin have been characterized in the marine environment. These include aldehydes in bacteria, tetrapyrroles in dinoflagellates, imidazolopyrazines including coelenterazine and luciferin of ostracods (vargulin), and more recently, the luciferin of *Odontosyllis,* a small polychaete^1,3,9,10^^.^ The most widespread luciferin is coelenterazine; biochemical assays have detected this molecule in nine phyla that display luminous species^3,11,12^. Moreover, two coelenterazine storage forms have been identified in several species, allowing better molecular stability of this luminous substrate^1,13,14^. The broad phylogenetic distribution of the imidazolopyrazine luciferins has led scientists to assume a potential dietary acquisition of these molecules through the food chain. This luciferin trophic acquisition has been experimentally demonstrated in several species. Vargulin, originating from copepods, has also been found to be the substrate for light emission in the teleost fishes *Porichthys notatus* and *Parapriacanthus ransonnetti* after consumption of either luminous ostracods or synthetic vargulin^15,16^^.^ Two populations of *P. notatus*, living in distinct regions, have been identified: one is luminous, while the other is non-luminous. The ability to emit light was linked to the presence or absence of the ostracod, *Vargula tsujii,* which was preyed upon by *P. notatus*^15^. More recently, in addition to the vargulin dietary acquisition, the first luciferase acquisition through a diet has been demonstrated in *P. ransonnetti*^16^. Coelenterazine trophic acquisition has been experimentally demonstrated in three phylogenetically distinct species: a shrimp (*Gnatauphausia ingens*), a jellyfish (*Aequorea victoria*), and a brittle star (*Amphiura filiformis*)^11,17,18^^.^ The first study to describe the coelenterazine trophic acquisition concerned the shrimp *G. ingens*^17^*.* A loss of luminous capability was observed from two months in captivity with a restricted coelenterazine diet. The shrimps rapidly recovered their capabilities when fed with luminescent prey^17^. Then, Haddock et al.^18^ studied the luminous capabilities of multiple generations of the jellyfish *A. victoria* in a controlled environment. This species is well known for its luminous system, a photoprotein containing coelenterazine called aequorin, which emits light in the presence of calcium^1,8^. This study showed that the jellyfish *A. victoria* regains its luminous capabilities only 8 h after a single ingestion of wild-caught luminous prey. This reacquisition was even faster after injecting synthetic coelenterazine directly into the jellyfish mesoglea. Despite the short time required to observe an increase in the luminous capabilities, the latter never reached the level of wild-caught *A. victoria*^18^.

The most recent study on the trophic acquisition of coelenterazine focused on the brittle star *A. filiformis*^11^*,* which inhabits soft sediments in the North and Mediterranean Sea^19^. This species has been studied to characterize its bioluminescence, including investigations into the regulation processes, photogenic sites, and the luminous system. This brittle star emits blue light at 472 nm owing to a coelenterazine-dependent luciferase, which is homologous to the luciferase of the cnidarian *Renilla reniformis*^11,20,21^*.* Physiological studies of light emission regulation revealed a cholinergic pathway involving muscarinic and nicotinic receptors in the *A. filiformis* bioluminescent flash production^22^. Light production mainly comes from the arm spines, where specific cells called photocytes are responsible for bioluminescence^23^. The inner part of the spine contains different cell types, including neural cells, cells secreting mucus (mucocytes), and two granular cells, which are the most abundant. The type I granular cells display large granules from 300 nm to 3 µm in diameter, while the type II granular cells enclose smaller granules from about 1 µm diameter comprising reticulated material, which were suspected to be the photogenic site, the photocytes^23^. Mallefet et al.^11^ went deeper into understanding the *A. filiformis* luminous system by demonstrating the need for a coelenterazine exogenous supply to maintain the brittle star's luminous capabilities. The natural luminous capabilities of brittle stars caught during the spring season have dropped significantly after five months in captivity with a coelenterazine-free diet. This loss of luminous capabilities was reversed after a single coelenterazine boost^11^.

The starting point of the present study was the dependency of *A. filiformis* luminous capabilities on a coelenterazine food supply^11^. As an infaunal species, *A. filiformis* extends two arms in the water column and feeds from the suspended materials (seston)^24,25^. Besides, seasonal blooms induce a massive increase of zooplanktonic species potentially consumed by *A. filiformis* during summer and fall, plankton community variation which might affect natural luminous capabilities during the season^26^.

Therefore, questions remain on (*i*) the seasonal variation of the initial luminous capabilities and subsequently on the depletion and reacquisition of luminous capabilities over seasons, (*ii*) the expression of the luciferase across depletion and induction periods, and (*iii*) the variation and localization of coelenterazine and its putative storage forms (*e.g.,* enol-sulfate coelenterazine, dehydrocoelenterazine). A long-term study was undertaken for more than a year; for each season, individuals were sampled and maintained in captivity to follow the temporal variation in *A. filiformis’*s luminous capabilities.

Brittle stars were fed either an unsupplemented or supplemented coelenterazine diet to confirm the trophic acquisition hypothesis and to monitor potential seasonal variations. For the first time, histological and cytological studies were performed to visualize the dynamic increase of the coelenterazine autofluorescent signal concomitantly with the luciferase expression within the photogenic sites after an exogenous supply of coelenterazine.

## Material and methods

### Seasonal sampling

*A. filiformis* (Müller, 1776) individuals were collected using an Eckman grab at a depth of 30–40 m in the Gullmarsfjord near the Kristineberg Marine Research Station (University of Gothenburg, Fiskebäckskil, Sweden) in August 2021, November 2021, January 2022 and May 2022. The brittle stars were carefully rinsed out of the mud, and intact specimens were placed in an aquarium with running seawater pumped directly from the adjacent fjord. *Amphiura chiajei* (Forbes, 1843) individuals were sampled using the same method.

### Monitoring the luminous capabilities

As demonstrated recently, the brittle star *A. filiformis* loses the ability to emit light without any exogenous supply of coelenterazine^11^. Nevertheless, the potential variation of the light emission could depend on the time of the year. Therefore, a long-term seasonal monitoring protocol was set up to monitor these potential variations. This strategy requires the monitoring for every season of (*i)* the natural luminous capabilities, (*ii)* the depletion, (*iii)* the induction, (*iv)* the depletion of the induced luminous capabilities, and (*v)* a second induction of luminous capabilities for fall and winter batches (Supplementary Fig. S1).

### Monitoring of the natural luminous capabilities

For each season, *A. filiformis* (n = 30) natural luminous capabilities were directly measured with KCl depolarization, acetylcholine (Ach) application, and coelenterazine and luciferase assays via the protocol described in the following sections.

### Dissection

Specimens were anesthetized by immersion in MgCl_2_ solution (3.5%) for 3 minutes^22,27^. Four arms were removed from the disc of each specimen and weighed. Two arms were placed in different tubes containing 500 µL of artificial seawater (ASW; 400 mM NaCl, 9.6 mM KCl, 52.3 mM MgCl_2_, 9.9 mM CaCl_2_, 27.7 mM Na_2_SO_4_, 20 mM Tris; pH 8.2) for KCl and Ach applications. The other two and the disc were frozen directly at -20 °C for biochemical assays. The fifth arm was kept as a backup tissue if necessary.

### Luminometric assays

Measurements of the light emission were carried out following Mallefet et al. (2020). An FB12 tube luminometer (Tirtertek-Berthold, Pforzheim, Germany) was kept in the dark room and calibrated using a standard 470 nm light source (Beta light, Saunders Technology, Hayes, UK). Light responses were recorded using FB12-Sirius PC Software (Tirtertek-Berthold). All data were standardized per unit of mass (g).

For KCl application, light emission was triggered by injecting 500 μl of KCl stock solution (400 mM KCl, 52.3 mM MgCl_2_, 9.9 mM CaCl_2_, 27.7 mM Na_2_SO_4_, 20 mM Tris; pH 8.2). For the Ach application, 500 μl of Ach stock solution (2 mM Ach, 52.3 mM MgCl_2_, 9.9 mM CaCl_2_, 27.7 mM Na_2_SO_4_, 20 mM Tris; pH 8.2) was injected to trigger light emission. The total amount of light emitted (Ltot) for both simulations was recorded and converted into quanta per gram of arm tissue (10^9^ q g^−1^).

For coelenterazine detection, one frozen arm or a disc was placed in an Eppendorf tube containing 200 μl of cold methanol and crushed with a micro-pestle. Then, 5 μl of the methanolic extract was placed into a tube filled with 195 μl of Tris buffer (20 mM Tris, 0.5 M NaCl; pH 7.4) and inserted in the luminometer. Afterward, 200 μl of *Renilla* luciferase solution with 4 μl of *Renilla* luciferase (Prolume Ltd., working dilution of 0.2 g l^–1^ in a Tris–HCl buffer 10 mM, NaCl 0.5 M, and BSA 1%; pH 7.4) diluted in 196 μl of Tris buffer was injected into the luminometer tube. The Ltot was recorded to calculate the coelenterazine content per gram of arm tissue (ng g^−1^), assuming that 1 ng of pure coelenterazine coupled with *Renilla* luciferase emits 2.52 × 10^11^ photons^1^.

For the luciferase assay, the other frozen arm was placed in an Eppendorf tube containing 200 μl of Tris buffer (20 mM Tris, 0.5 M NaCl; pH 7.4) and crushed with a micro-pestle until a homogenized extract was obtained. Then, 20 and 40 μl of the extract were placed in two tubes with 180 and 160 μl Tris buffer, respectively. Each tube with the diluted luciferase solution was placed in the luminometer, and a solution containing 5 μl of 1/200 stock of coelenterazine (1OD in cold methanol at 430 nm; Prolume Ltd, USA) diluted in 195 μl of Tris buffer was injected. Two measures of maximum light emission (Lmax) were recorded and averaged to calculate the maximal light decay rate corresponding to the luciferase activity^1^, expressed in 10^9^ quanta g^−1^ s^−1^.

### Luminous capabilities for long-term monitoring

For each season, brittle stars were transported to the Marine Biology Laboratory at the Université catholique de Louvain (Belgium). Then, 400 specimens were isolated in closed-circuit aquariums filled with artificial recirculating seawater (35 salinity, low nitrate, pH 8.2) containing 40 mm diameter PVC rings with two brittle stars in each. During the two-year captivity experiment, the room temperature was constantly adjusted to the temperature recorded at the fjord bottom) to mimic seasonal temperature variations (https://www.weather.mi.gu.se/kristineberg/en/data.shtml). Animals were captive for several years with a constant photoperiod (12 h:12 h light: dark).

The luminous capabilities of the summer batch were monitored from August 2021 to December 2022. Then, the luminous capabilities of the fall batch were measured from November 2021 to March 2023. The luminous capabilities of the winter batch were monitored from January 2022 to May 2023. Finally, the luminous capabilities of the spring batch were recorded from May 2022 to September 2023.

### Depletion protocol

For eight months, coelenterazine-free food (Hikari Plankton) was provided to the collected brittle stars once a week. Every month, in addition to KCl and Ach applications to monitor the depletion of natural luminous capabilities, coelenterazine, and luciferase assays were performed as described above. A second depletion was monitored for each seasonal batch.

### Induction protocol

After the 8-month depletion period (depletion 1; Dep 1), 330 brittle stars received a single dose of coelenterazine-supplemented pellets (Nutra HP 1.0 with a mean value of 280 ng of coelenterazine per gram of food) (induction 1; I1). Luminous capabilities were tested every week over one month after the exogenous supply. Then, the luminous capabilities were followed monthly (depletion 2; Dep 2). Due to the requested specimen number and time-consuming protocol, only two successive induction protocols were performed for the fall and winter seasons (induction 2; I2). In contrast, one induction event was done for the spring and summer brittle star batches. Due to limited animal numbers, the depletion 2 in winter was reduced to six months (Supplementary Fig. S1).

### Coelenterazine storage forms assays

To unveil the potential use of coelenterazine storage forms (*i.e.,* enol-sulfate coelenterazine and dehydrocoelenterazine) by the brittle star *A. filiformis,* a similar protocol to that used by Shimomura was followed^1^. Briefly, for the enol-sulfate assay, 10 µL of the methanolic extract was added to 100 µL of HCl 0.5 M, heated at 95 °C for 1 min, cooled down on ice water, then neutralized with a small amount of solid NaHCO_3_. Then, 90 µL of Tris buffer (20 mM Tris, 0.5 M NaCl; pH 7.4) was added to the tube and placed in the luminometer. Commercial *Renilla* luciferase solution was prepared for the coelenterazine assay and injected into the luminometer tube. For the dehydrocoelenterazine assay, 0.5 mg of solid NaBH_4_ was added to 10 µL of the methanolic extract, rested for 5 min, and then placed in the luminometer. Aside from this, 4 µL of the commercial luciferase was diluted in 400 µL of Tris buffer (20 mM Tris, 0.5 M NaCl; pH 7.4) and injected into the luminometer tube. For both assays, the Ltot was recorded. The values obtained with both assays correspond to the sum of the free coelenterazine content and the storage forms. The recorded Ltot for the free coelenterazine was subtracted from the Ltot obtained with the storage form assays1 to obtain the storage form amounts. These assays were performed on depletion and induction periods on 12 specimens each.

### Histological visualization during short-term induction monitoring

Precise monitoring of the luminometric measurements before induction and from 3 to 384 h post-induction was undertaken on 48 brittle stars. This approach combines *(i)* characterization of the spine structure, (*ii)* coelenterazine histological visualization, (*iii)* immunodetection of luciferase, and (*iv)* luminometric measurements of the coelenterazine content and the luciferase activity in arm tissue.

### Spine morphological structure

A transmission electron microscopy protocol was applied for the ultrastructure observation of the photogenic structure and associated cells among the arm spine of *A. filiformis*^23^*.*

Arms portions from three *A. filiformis* specimens were fixed in a glutaraldehyde solution (3% C_5_H_8_O_2_, 0.1 M C_2_H_6_AsNaO_2_, 0.27 M NaCl; pH 7.8) for three hours, then rinsed in a cacodylate buffer (0.2 M C_2_H_6_AsNaO_2_, 0.31 M NaCl; pH 8.4). Samples were then post-fixed in osmium tetroxide (1% OsO_4_, 0.1 M C_2_H_6_AsNaO_2_, 0.27 M NaCl; pH 7.8) during one hour and rinsed again in a cacodylate buffer. After the last wash, the pieces were decalcified for 30 h in a 1:1 mixture of 2% ascorbic acid and 0.3 M NaCl^28^ and then progressively dehydrated in a graded ethanol concentration series. The samples were embedded in Spurr’s resin (EM0300, Merck, Darmstadt, Germany) following Delroisse et al.,^23^. Semithin sections of 400–500 nm were obtained using a Leica Ultracut UCT ultramicrotome (Leica microsystems, Wetzlar, Germany) and stained with a 1:1 mixture of 1% methylene blue and 1% azure II^29^. Semithin sections were observed under a stereomicroscope (Zeiss Axio Scope A1, Zeiss, Oberkochen, Germany) coupled with an Axiocam 305 color camera (Zeiss). Ultrathin sections of 90 nm were obtained using a Leica Ultracut UCT ultramicrotome (Leica) and delicately placed on copper grids. Sections were subjected to uranyl acetate contrast solution (0.18 M uranyl acetate solution: ethanol (2:1)) in dark chambers for 45 min, followed by a lead citrate bath (0.08 M Pb(NO_3_)_2_, 0.12 M Na_3_C_6_H_5_O_7_, 0.16 M NaOH) for 4.5 min, and left to dry. Sections were observed in a transmission electron microscope Zeiss Leo 906E (Zeiss) coupled with a Jenoptik camera (Jenoptic ProgRes CFcool, Iéna, Germany) and micrographed.

### Dissection and luminometric measurements

After the exogenous supply of coelenterazine, animals were anesthetized by immersion in MgCl_2_ solution (3.5%) for 3 minutes^22,27^. For each time post-induction (t = 3, 24, 48, 69, 192, 240, and 384 h), each specimen (n = 6) arms were dissected and weighed. One arm was placed in a tube containing ASW for the KCl application to validate the luminous capability reacquisition after induction. One arm was placed in Eppendorf with MgCl_2_ for confocal observation (Stellaris 8 Falcon, Leica, Germany). One arm was fixed in phosphate buffer saline (PBS: 123 mM NaCl, 2.6 mM KCl, 12.6 mM Na_2_HPO_4_, 1.7 mM KH_2_PO_4_, pH 7.4) containing 4% paraformaldehyde. The coelenterazine and luciferase content was coupled with luminometric assays for which the other two arms and the disc were frozen directly at -20 °C, and biochemical assays were performed as described above.

### Coelenterazine histolocalization

The 1/200 stock of coelenterazine (1OD in cold methanol at 430 nm; Prolume Ltd #303, USA) presents a native green autofluorescence ($\lambda$ max excitation = 489 nm and $\lambda$ max emission = 545 nm; Prolume Ltd, USA). The arm kept in an Eppendorf with MgCl_2_ was directly mounted in glycerol (Glycerol®, Sigma) to observe *in toto* the coelenterazine-signal with a confocal microscope (Leica,Stellaris 8 Falcon, Germany). Three controls were used: (*i*) wild-caught *A. filiformis*, (*ii) A. filiformis* maintained in captivity for 22 months fed with a coelenterazine-free diet, and (*iii) A. chiajei*, a non-luminous sympatric brittle star, fed with coelenterazine-supplemented pellets.

### Luciferase immunolocalization

The fixed arms were rinsed three times in PBS and then blocked for 2 h in PBS with 6% bovine serum albumin (BSA) and 2% Triton X100 at RT. *Renilla* luciferase antibody (GTX125851, Genetex)^20,23^ was diluted 1:500 in PBS containing 6% BSA and 1% Triton X100. After overnight incubation (at room temperature), arms were rinsed at least six times 30 min in PBS-1% Triton X100 and then incubated in a 1:500 dilution of Alexafluor 594 conjugated antirabbit (A11037, Thermofisher Invitrogen) in PBS containing 1% Triton X100 and 6% BSA. After incubation (overnight, RT), arms were rinsed six times 30 min with PBS-1% Triton X100. Arms were mounted in Mowiol (Mowiol® 4–88, Sigma) and examined using a confocal microscope (Stellaris 8 Falcon). As for the coelenterazine localization, three controls were performed with wild-caught and depleted *A. filiformis* and with *A. chiajei* fed with coelenterazine-supplemented pellets. In addition, a control was performed by omitting the primary antibody to ensure the absence of unspecific binding of the secondary antibody.

### Statistical analysis

All the statistical analyses were performed with R Studio (version 2023.06.1, 2022, Posit Software, USA). Variance normality and equality were tested using the Shapiro–Wilk test and Levene’s test, respectively. When these parametric assumptions were met, an ANOVA was coupled with the Tuckey test. When log transformation does not allow reaching normality and homoscedasticity, non-parametric Kruskal–Wallis ANOVA and Wilcoxon sum rank tests were used.

This statistical approach was performed to compare wild-caught luminometric measurements from each season. These tests were also used during the long-term monitoring to perform comparisons between (*i*) day 0 and depletion 1, (*ii)* day 240 and induction 1, (*iii)* day 268 and depletion 2, and (*iv*) for fall and winter seasons, day 478 and day 448 to induction 2, respectively. Finally, non-parametric Kruskal–Wallis ANOVA and a Wilcoxon sum rank test were used to assess the significant difference between hour 0 and post-induction times during the short-term induction monitoring. Each difference was considered to be significant at a minimum P-value < 0.05. Values were graphically illustrated with mean and standard error of the mean (s.e.m). Additionally, Principal Component Analysis (PCA) was performed each season to assess correlations between luminometric parameters.

## Results

### Seasonal monitoring of the luminous capabilities

**Figure 1:**
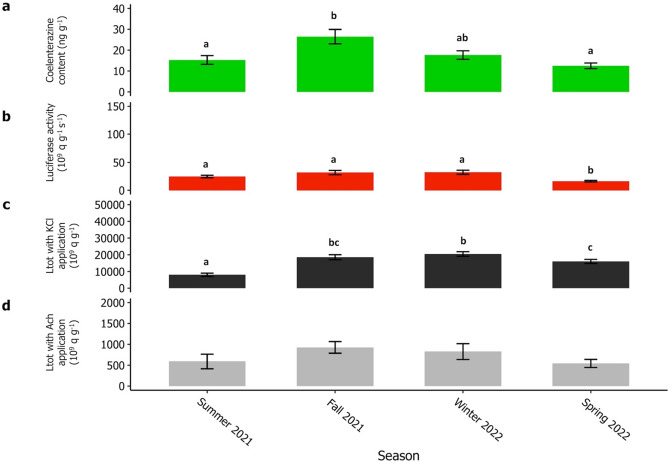
Seasonal monitoring of the natural luminous capabilities. Luminometric parameters were recorded on wild-caught *A. filiformis* specimens during (**a**) summer 2021, (**b**) fall 2021, (**c**) winter 2022, and (**d**) spring 2022. For each season, green bars correspond to the coelenterazine content (ng g^−1^), red bars correspond to the luciferase activity (10^9^ q g^−1^ s^−1^), black bars correspond to the Ltot with KCl application (10^9^ q g^−1^), and grey bars correspond to the Ltot with Ach application (10^9^ q g^−1^). Values are expressed as mean ± s.e.m. Different lettering indicates statistical differences (n = 30). According to the parametric assumptions, one-way with ANOVA coupled with the Tuckey test or with Kruskal–Wallis ANOVA coupled with Wilcoxon sum rank. Statistical differences were highlighted with a *P*-value < 0.05.

The luminometric measurements performed on wild-caught brittle stars (*i.e.,* coelenterazine content, luciferase activity, light emissions after KCl and Ach applications) were recorded in Sweden at each season (n = 30) (Fig. 1). First of all, *A. filiformis* appeared to be able to emit light throughout the year, even if its luminous capabilities varied among seasons. Then, two different patterns emerged among the four luminometric measurements. Firstly, the coelenterazine content in *A. filiformis* arms and the light emission after KCl application were significantly higher in fall 2021 and winter 2022 (Fig. 1). The highest coelenterazine concentration was measured in fall 2021 with an average value of 26.48 ± 3.44 ng g^−1^. The maximal Ltot value was observed in winter 2022 (20,481 ± 1389 10^9^ q g^−1^). Despite the absence of significant seasonal differences in the Ltot after the Ach application, a similar trend appeared, with the highest Ltot value in winter 2022 (826 ± 189 10^9^ q g^−1^). Secondly, the luciferase activity remained stable along seasons except in spring 2022, where the mean value was significantly lower (16.3 ± 1.3 10^9^ q g^−1^ s^−1^) (Fig. 1). Overall, the summer season displayed a tendency of lower values for all the measured luminometric parameters (Fig. 1). Supplementary Table S1 summarizes the mean and s.e.m values from coelenterazine and luciferase assays and KCl and Ach applications with the statistical test and the associated p-value for each season.

### Long-term monitoring of the luminous capabilities

#### Summer 2021

**Figure 2:**
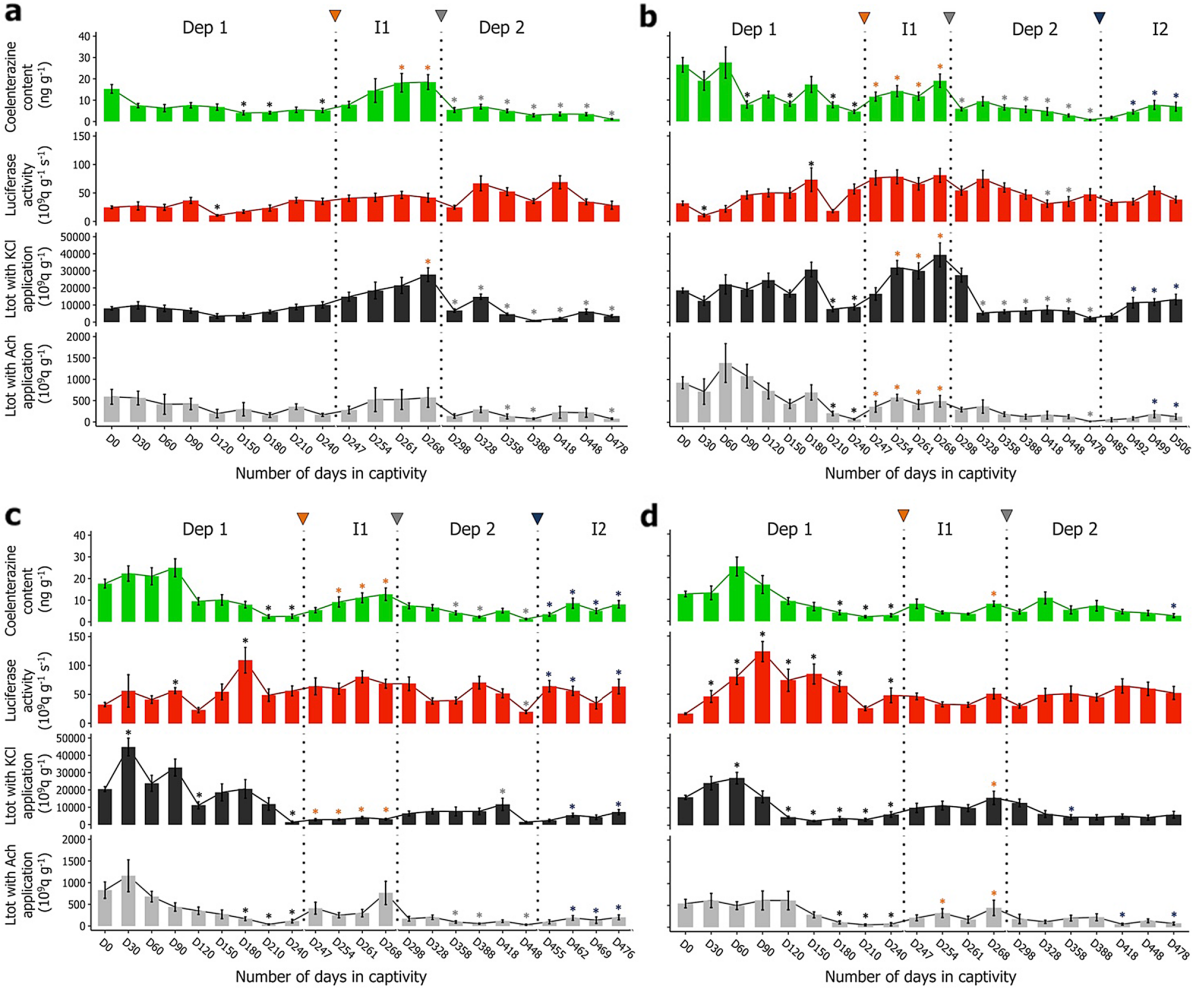
Long-term monitoring of the luminous capabilities for each season. Luminometric parameters recorded during long-term monitoring for individuals initially sampled during (**a**) summer 2021, (**b**) fall 2021, (**c**) winter 2022, and (**d**) spring 2022. For each season, green bars correspond to the coelenterazine content (ng g^-1^), red bars correspond to the luciferase activity (10^9^ q g^−1^ s^−1^), black bars correspond to the Ltot with KCl application (10^9^ q g^−1^), and grey bars correspond to the Ltot with Ach application (10^9^ q g^−1^). For each season, individuals were maintained with a coelenterazine-free diet till D240 (depletion 1), followed by a single boost of coelenterazine (orange arrowhead), and parameters were recorded for four weeks (induction 1, grey arrowhead), and then again monthly (depletion 2). A second single boost of coelenterazine (induction 2, blue arrowhead) was given to the individuals initially sampled in fall 2021 and winter 2022. Again, the parameters for the second induction period were monitored weekly (**b**, **c**). The double bars indicate a change of time step from monthly to weekly measurements. Dep 1: First depletion period; I1: First induction period; Dep 2: Second depletion period; I2: Second induction period. Values are expressed as mean ± s.e.m. Statistical differences (P-value < 0.05) are indicated by black asterisks (*) between day 0 and depletion 1 measurements; orange asterisks (*) between day 240 and induction 1 measurements; grey asterisks (*) between day 268 and depletion 2 measurements; and blue asterisks (*) between day 478 (for fall) or day 448 (for winter) and induction 2 measurements. According to the parametric assumptions, either one-way ANOVA and Tuckey test or Kruskal–Wallis ANOVA and Wilcoxon sum rank test were performed.

The mean coelenterazine content measured in *A. filiformis* arm tissue decreased progressively during the initial depletion phase, reaching significantly lower values after 150, 180, and 240 days (4.1 ± 1.0; 4.2 ± 0.6; 5.2 ± 1.0 ng g^−1^ respectively; Fig. 2a). Following a single boost of coelenterazine (day 244), coelenterazine content increased, reaching a significantly higher value than day 240 at day 261 (18.1 ± 4.4 ng g^−1^; Fig. 2a). Subsequently, during the second depletion phase, all coelenterazine content measurements dropped significantly compared to the end of the induction phase (day 268; Fig. 2a). Globally, coelenterazine content decreased after long-term maintenance in captivity (Fig. 2a).

The luciferase activity measured in arm tissue varied unevenly over the captivity period with or without any boost of coelenterazine in the brittle star diet (Fig. 2a). The lowest luciferase activity value was recorded after 120 days of captivity (10.6 ± 1.3 10^9^ q g^−1^ s^−1^; Fig. 2a) during the depletion phase, being the only statistically different value.

The Ltot after KCl application remained stable during the first depletion period till day 240. After the exogenous supply of coelenterazine (day 244), the Ltot with KCl application increased continuously till reaching a significantly higher value at day 268 (27,782 ± 4,036 10^9^ q g^−1^; Fig. 2a). As for the coelenterazine content, the Ltot values after the induction phase (day 268) dropped significantly till the end of the experiment (Fig. 2a).

Analysis of acetylcholine response revealed no statistical differences either during the depletion or induction periods (Fig. 2a). Conversely, differences were observed during the second depletion phase (Fig. 2a), with values statistically distinct from days 268 at 358, 388, and 478 days (129 ± 56; 75 ± 17; and 75 ± 17 10^9^ q g^−1^, respectively).

### Fall 2021

The mean values for the coelenterazine content during the first depletion of the fall season decreased gradually with statistical differences compared to day 0 at days 120, 180, 210, and 240 (Fig. 2b). The lowest value encountered during this depletion phase was at day 240 (4.6 ± 0.8 ng g^−1^; Fig. 2b). After the first coelenterazine supply (day 244; Fig. 2b), values significantly increased all along the induction month. During the second depletion period, coelenterazine contents dropped statistically, except at day 328 (Fig. 2b). A second boost of coelenterazine was performed on day 482, and a significant increase in coelenterazine content appeared after only ten days (Fig. 2b).

Globally, measured luciferase activities presented fluctuating values throughout the long-term monitoring of the fall season. During the first depletion, two values were significantly different from the natural ones (day 0); one at day 30 (10.8 ± 2.5 10^9^ q g^−1^ s^−1^) presented a significantly lower value, while the other, at day 180 (73.3 ± 20.7 10^9^ q g^−1^ s^−1^), had a significantly higher value (Fig. 2b). Similarly, only two values displayed significant lower values during the second depletion phase, at day 418 and 448 (31.0 ± 6.5, and 35.0 ± 8.6 10^9^ q g^−1^ s^−1^, respectively; Fig. 2b).

Measurements of Ltot after KCl application fluctuated during the first depletion period, reaching significantly lower values at day 210 and 240 (Fig. 2b). The first exogenous supply of coelenterazine induced a constant rise of the light emission, displaying a significant difference from day 240 after ten days post supply till the end of the induction month (Fig. 2b). A rapid decrease during the second depletion phase was observed, presenting statistically lower values from days 328 to 478. The lowest value was measured after 478 days (2424 ± 886 10^9^ q g^−1^; Fig. 2b). As for the first induction, the second boost of coelenterazine induced an increase of the Ltot values, statistically different only ten days after the exogenous supply (Fig. 2b).

Ltot triggered by Ach application significantly dropped at days 210 and 240 of captivity without any exogenous supply of coelenterazine. A significant increase was observed during the first induction period, from day 247 to 268 (Fig. 2b). Subsequently, a significant decrease was measured for day 478 (21 ± 7 10^9^ q g^−1^) during the second depletion phase (Fig. 2b). The second induction with an exogenous boost of coelenterazine presented significant differences from day 478 at days 499 and 506 (190 ± 82, and 134 ± 64 10^9^ q g^−1^; Fig. 2b).

### Winter 2022

During the first depletion phase, coelenterazine content showed statistically lower values from the natural ones at days 210 and 240 (Fig. 2c). Once again, ten days after the first coelenterazine exogenous supply, coelenterazine content significantly increased all along the induction month. The coelenterazine content decreased significantly during the second depletion on days 358, 388, and 448. The lowest value appeared at the end of this depletion phase precisely at day 448 (1.3 ± 0.3 ng g^−1^; Fig. 2c). Coelenterazine content raised significantly directly after the second exogenous supply (Fig. 2c).

As for the previous season batches, the luciferase activity measured in specimens sampled in winter 2022 fluctuated throughout the captivity period, whether or not the food was supplemented with coelenterazine, occasionally presenting some statistical differences (*i.e.,* days 120, 180, 448, 455, 462, 476; Fig. 2c).

The Ltot determined after KCl application fluctuated at the beginning of the captivity period with a maximum at day 30 (44,757 ± 5,131 10^9^ q g^−1^; Fig. 2c) and significantly dropped at day 120 and 240 (11,175 ± 2039, and 1,410 ± 663 10^9^ q g^−1^, respectively; Fig. 2c). Even if presenting relatively low values, the exogenous coelenterazine supply induced a significant increase of Ltot during the first induction period (Fig. 2c). The Ltot values remained stable during the second depletion except at day 418 (11,653 ± 3547 10^9^ q g^−1^) which displayed a significantly higher than day 268 (Fig. 2c). During the second induction the Ltot values increased significantly at days 462 and 476 (Fig. 2c).

The Ltot values after Ach application significantly dropped among the last three months of the first depletion period (*i.e.* days 180, 210, 240; Fig. 2c). Even if no statistical differences occurred during the first induction period, values tended to increase. Then, during the second depletion phase, significant drops were observed at days 358, 388, and 448 (Fig. 2c). Finally, a statistical increase of Ltot values was observed ten days after the second exogenous supply of coelenterazine (Fig. 2c).

### Spring 2022

For the coelenterazine content measured in individuals from the spring 2022 collection, values stayed stable during the first half of the depletion period, then decreased gradually, reaching significantly lower values from day 180 to day 240. Except for the last week of the induction period, coelenterazine content values appeared not significantly different from day 240 (Fig. 2d). During the second depletion phase, the coelenterazine content dropped slowly until reaching a significantly lower value at the end of the experiment (day 478; 2.5 ± 0.9 ng g^−1^; Fig. 2d).

During the first depletion period, except at day 210, all the luciferase activity values were significantly higher than the natural ones. For the remaining time of the experiment, the activity values oscillated from 25.5 to 64.2 10^9^ q g^−1^ s^−1^(Fig. 2d).

Although a significant increase of the Ltot value after KCl application measured at day 60, subsequent weeks of the first depletion were characterized by significant decreases from days 180 to 240 (2417 ± 310, 3937 ± 1083, 3127 ± 845 10^9^ q g^−1^, respectively; Fig. 2d). During the induction period, Ltot values raised until reaching a significantly higher value four weeks after the coelenterazine boost (day 268: 15,695 ± 3821 10^9^ q g^−1^). The only significantly lower Ltot values appeared at day 358 (4635 ± 1398 10^9^ q g^−1^; Fig. 2d).

As for the coelenterazine content, Ltot values after Ach application displayed significantly lower values at days 180, 210, and 240 of the first depletion period (Fig. 2d). During the induction phase, the Ltot values triggered by Ach showed a significant increase in comparison with day 240 at days 254 and 268 (323 ± 101, and 444 ± 176 10^9^ q g^−1^, respectively). Subsequently, during the second depletion phase, a slight decrease was observed till reaching a statistically lower value at days 418 and 478 (69 ± 22 and 85 ± 32 10^9^ q g^−1^, respectively).

Supplementary Table S2 presents all the mean and s.e.m values from coelenterazine and luciferase assays and KCl and Ach applications, along with the sample size, statistical test, and associated p-value.

According to the PCA results (Supplementary Fig. S2), luciferase activity showed no correlation with the three other luminometric parameters (*i.e.,* coelenterazine content and Ltot value for KCl or Ach applications). Conversely, positive correlations were underlined between the three former parameters.

### Coelenterazine storage forms

Following Shimomura protocols^1^, storage forms assays (n = 12) did not detect the presence of coelenterazine storage derivatives, dehydrocoelenterazine, and enol sulfate coelenterazine. All recorded values were lower than the recorded coelenterazine amounts, whether the brittle stars were fed with coelenterazine-supplemented food or not (Supplementary Fig. S3).

### Spine morphological structure

**Figure 3:**
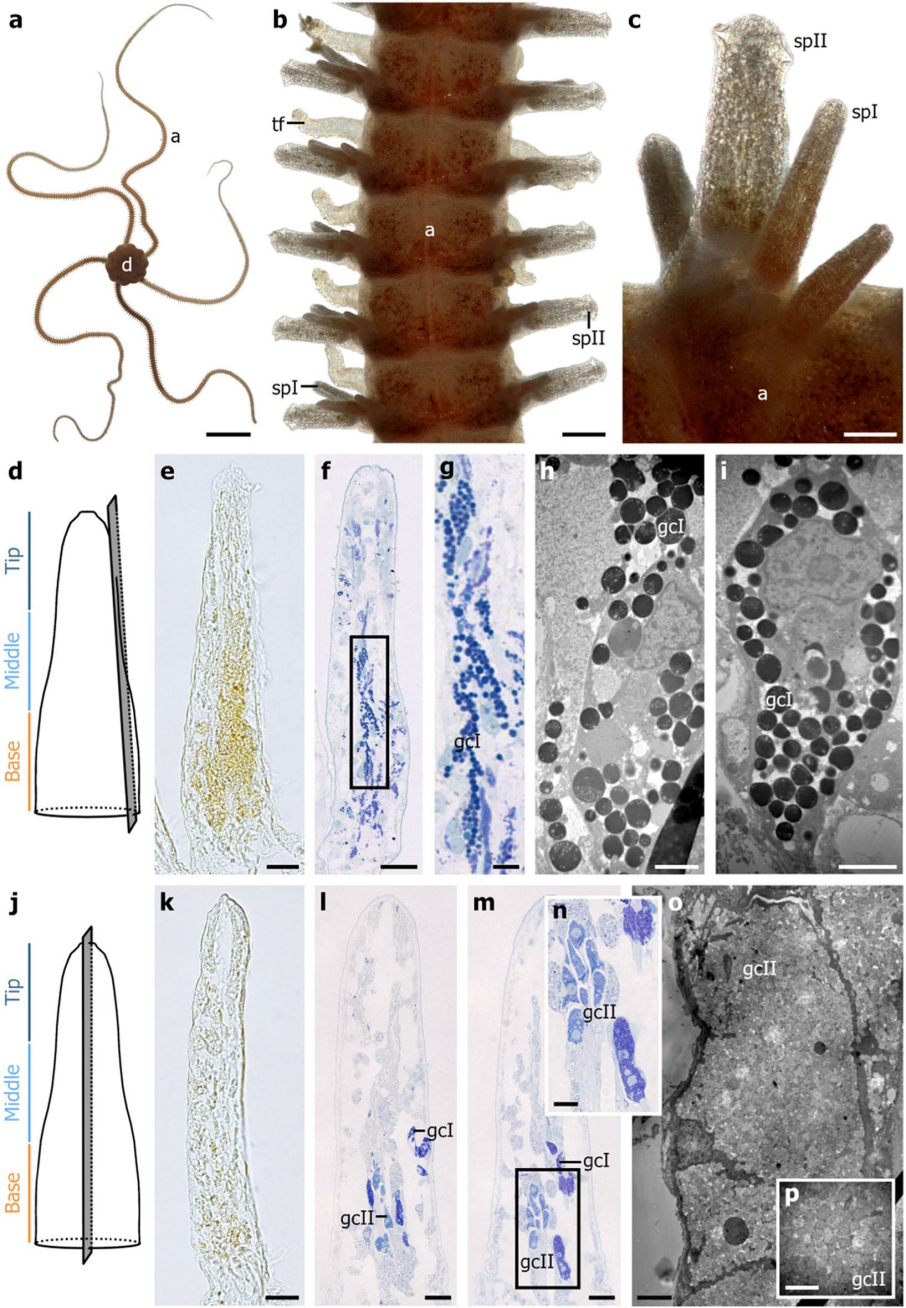
Spine morphological structure of *Amphiura filiformis*. (**a**) Aboral view of the brittle star. (**b**) Aboral view of an arm showing five successive segments. (**c**) Detail of the two spine morphotypes worn on each arm segment. (**d**) Scheme of the cutting plane (grey rectangle) within the spine's outer tissues. (**e**) Unstained paraffin longitudinal section of the spine outer tissues showing yellow-orange pigments. (**f**) Semithin longitudinal section of the spine outer tissues under 1:1 mixture of methylene blue and azure II staining. (**g**) Close-up of the type I granular cells in f. (**h**, **i**) TEM micrographs of the type I granular cells. (**j**) Scheme of the cutting plane (grey rectangle) within the spine inner tissues for k-n. (**k**) Unstained paraffin longitudinal section of the spine inner tissues. (**l**, **m**) Semithin longitudinal section of the spine inner tissues under 1:1 mixture of methylene blue and azure II staining. (**n**) Close-up of the type II granular cells. (**o**) TEM micrograph of a type II granular cell. (**p**) Close-up of the granules enclosed in type II granular cells. a: arm, d: disc, gcI: type I granular cells, gcII: type II granular cell, spI: type I spine, spII: type II spine, tf: tube feet. Scale bars: a = 1 cm; b = 250 µm; c = 100 µm; e, f, k, l, m = 20 µm; g = 5 µm; h, i, o = 2 µm; n = 10 µm; p = 1 µm.

The arm spines were inserted on each of the 200 articulated segments, and two spine morphotypes were easily recognizable with digital microscopy (Fig. 3a, b, c). According to Delroisse et al.,^23^, the tapered spines were classified as the type I spine, while the lateral-most spine characterized by a rectangular shape was identified as the type II spine (Fig. 3b, c). The general spatial organization of the different cell types, described below, is the same for both spine morphotypes. In unstained paraffin sections, the spine outer tissue displayed clusters of yellow-orange cells at the basal and middle areas of the spine (Fig. 3d, e). On semithin sections, dark blue granules were observed in the same regions of the spine (Fig. 3f, g). In transmission electron microscopy, these spherical granules, ranging from 0.5 to 1.5 µm in diameter, appeared to be electron-dense (Fig. 3h, i).

In the spine's inner tissues, a slight yellow-orange coloration restricted at the base of the spine was observable on paraffin sections (Fig. 3j, k). In semithin middle spine longitudinal sections, anecdotal observations of isolated type I granular cells were pinpointed at the base and middle of the spine, specifically at the spine edge, closed to the cuticle (Fig. 3l, m). Moreover, type II granular cells were localized at the central basal part of the structure (Fig. 3l, m, n). The formers were visualized by mid-blue stained cells forming large clusters at the base of the spine (Fig. 3l, m, n). The same cells were spotted under a transmission electron microscope with notable spherical granules (500 nm wide) characterized by fibrillar appearance and moderate electron density (Fig. 3o, p). The latter, described as photocytes by Delroisse et al.,^23^, presented a body cell size between 20 and 30 µm^23^.

### Histological detection of coelenterazine and luciferase

**Figure 4:**
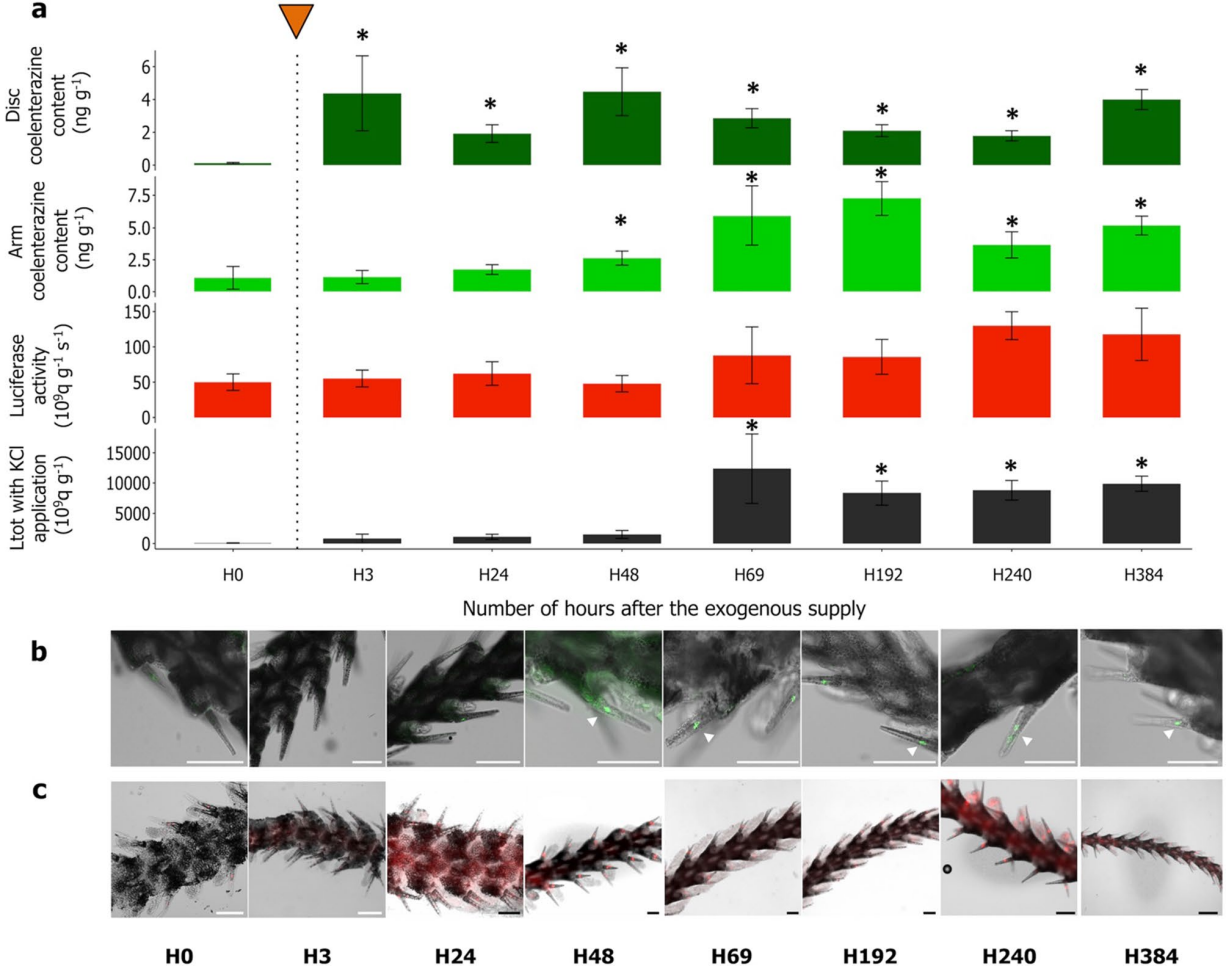
Monitoring the luminometric parameters, coelenterazine autofluorescence, and luciferase immunodetection in the arm’s spine before and after induction. (**a**) Luminometric parameters were recorded after a single boost of coelenterazine over 384 h. Green bars correspond to the coelenterazine content (ng g^−1^) in arms tissue, dark green bars correspond to the coelenterazine content (ng g^−1^) in the disk, red bars correspond to the luciferase activity (10^9^ q g^−1^ s^−1^), and black bars correspond to the Ltot with KCl application (10^9^ q g^−1^). Values are expressed as mean ± s.e.m. Asterisks indicate statistical differences between measurements performed before (H0) and after a boost of coelenterazine (n = 6). According to the parametric assumptions, either one-way ANOVA and Dunnett’s multiple comparisons test or Kruskal–Wallis ANOVA and Dunn multiple comparisons test comparisons test were performed. Statistical differences were highlighted with *P-value < 0.05. (**b**) Following the autofluorescence signal (green; white arrowhead) within the arm’s spine before and after an exogenous supply of coelenterazine. (**c**) Immunodetection (red) of luciferase expression within the arm’s spine before and after an exogenous supply of coelenterazine. Scale bar = 100 µm.

Luminometric measurements and morphological histological approaches were preliminary steps essential to visualizing the luminous components within the photogenic sites. Unfixed *A. filiformis* luminous arms in natural condition presented bright green autofluorescent dots under excitation at 489 nm at the level of the spines’ bases (Supplementary Fig. S4). The monitored green autofluorescent wavelength corresponded to the green autofluorescent wavelength of the native coelenterazine (1 OD in cold methanol at 430 nm; Prolume Ltd, USA). After 22 months with coelenterazine free-diet (H0), complete depletion of the brittle star's luminous capabilities and the disappearance of those green autofluorescent dots (Fig. 4a, b) were observed. Reacquisition of the greenish autofluorescence signals appeared concomitantly with increased coelenterazine contents in the arms tissues during induction phases (Fig. 4a, b). The luminometric measurements performed in the first hours post-induction were essential to characterize the reacquisition process speed and its histological consequences precisely. Only 3 h after the exogenous supply, the coelenterazine content increased significantly in the disc. Then, at 24 h post-induction, the coelenterazine content raised significantly in the arm’s tissue. Finally, the KCl Ltot was significantly enhanced 69 h after the coelenterazine boost. All the mean and s.e.m values from coelenterazine and luciferase assays and KCl applications are summed up with the sample size, statistical test, and the associated p-value in Supplementary Table S3.

Conversely, luciferase immunolabelling underlined a constant expression of this enzyme at the spine level all along the experiment, either during induction or depletion phases (Fig. 4c). This result was consistent with the stable luciferase activity monitored either through the long-term study (Fig. 2) or the focus on an induction phase (Fig. 4a). Both, the green autofluorescent dots and the luciferase immunodetection (Fig. 4b, c) were always co-localized with cells at the spine bases (Fig. 3n, o, p). The re-apparition of the green autofluorescence was visible after 48 h post-feeding. The same results were observed in the arm tips, presenting a green autofluorescence lost after luminous capabilities depletion. At the same time, the greenish autofluorescent dots reappeared to be expressed after an exogenous supply of coelenterazine (Supplementary Fig. S5). Similarly, arm tip luciferase expression was immunodetected during both depletion and induction phases, underlying a constant expression of this enzyme (Supplementary Fig. S5). Luminometric measurements control performed on *A. chiajei* are displayed in Supplementary Fig. S6. The control performed in the non-luminous sympatric species, *A. chiajei,* did not show any fluorescence at the spine or arm tip level, or did any immunodetection of luciferase (Supplementary Fig. S6). Control with omitting primary antibodies did not reveal immunolabeling (Supplementary Fig. S7).

## Discussion

The blue luminescence of the European burrowing brittle star *A. filiformis* was confirmed to be specifically dependent on the coelenterazine-dependent luciferase homologous to the *Renilla* luciferase^11,20^. Mallefet et al.^11^ were the first to maintain a luminous echinoderm in captivity for several years to monitor its luminescence. They experimentally demonstrated the trophic acquisition of the luminous capability in the brittle star *A. filiformis*^11^. As a suspension feeder, this infaunal species must feed on seston containing coelenterazine to maintain its luminous capabilities. Shimomura was the first to detect coelenterazine in luminous or non-luminous species and spotted coelenterazine in the plankton mixture^30,31^. Years later, planktonic species such as *Metridia pacifica*, *Bolinopsis infundibulum*, and *Mnemiopsis leidyi* were demonstrated to de novo synthesize this luciferin^32,33^. However, several studies have shown that planktonic communities shift in the Gullmarsfjord across seasons^26,34,35^. This phenomenon, coupled with the dependency on the exogenous supply of coelenterazine, raised the question of whether *A. filiformis* can produce light throughout the year.

Based on current data, it has been observed that *A. filiformis* does not possess any storage forms of coelenterazine, unlike other bioluminescent species that use such forms^13,14^. Enol sulfate coelenterazine has been found in the liver and light organs of *Gymnoscopelus braueri*, and squid species such as *Watasenia scintillans, Eucleoteuthis luminosa,* or *Symplectoteuthis oualaniensis* possess the dehydrocoelenterazine form^13,36^. These modified forms serve to stabilize and limit the oxidation of coelenterazine oxidation^14,37,38^.

The seasonal monitoring showed that *A. filiformis* can emit light throughout the year, with lower luminous capabilities in summer. The lower values of luminous capabilities during summer could be linked with the brittle star reproduction period. A recent study highlighted the presence of coelenterazine in male and female rape gonads^39^. Regardless of the ecological functions of luminescence, the brittle star *A. filiformis* may allocate more resources to gonad production than light emission during summer. The lower luminous capabilities during the reproductive period contradict observations made on another brittle star, *Amphipholis squamata,* which emits brighter light during winter, coinciding with its reproductive period^40^*.* Finally, coelenterazine storage within the gonads during summer might reduce coelenterazine availability in arm tissue.

The present seasonal, long-term monitoring highlighted the complexity and variability inherent to luminometric long-term monitoring. Luminometric measurements confirm, to a greater or lesser degree, a loss of *A. filiformis'* luminous capabilities when individuals were fed a coelenterazine-free diet. This observation attests to the inability of *A. filiformis* to de novo synthesize the coelenterazine substrate essential for the chemistry of its blue luminescence^11^. A hypothesis of a continuous coelenterazine supply in the *A. filiformis* diet throughout the year could be assumed according to our result. During the depletion of the natural luminous capabilities, all the luminometric parameters follow the same decreasing tendency except for the luciferase activity, which is stable over time. Irrespective of the sampling season, Mallefet et al. made the same observation for a singular batch of *A. filiformis*^11^.

For all seasonal batches, a single boost of coelenterazine elicits the reacquisition of luminous capabilities. The exogenous supply of coelenterazine administrated at each seasonal batch leads to a significant reacquisition of luminous capabilities for all batches. Nevertheless, the trophic reacquisition seems less efficient for the induction performed in November 2022 and January 2023 for the winter and spring batches. The efficiency of the induction protocol might be related to the seasons occurring during the induction protocol. Mallefet et al.^11^performed an efficient induction protocol and showed quick trophic reacquisition after an exogenous supply of coelenterazine in July. The *A. filiformis’* food intake could be limited during winter, which could be related to slowing the animal metabolism. A similar trophic dependency of coelenterazine was demonstrated in a shrimp and a jellyfish^17,18^. Both species maintained in captivity with a coelenterazine-free diet have lost their ability to produce light. This luminous status was regained when individuals were fed with luminous preys containing coelenterazine^17,18^. Nevertheless, none of these studies investigated the potential seasonal variation during the reacquisition of luminous capabilities.

Our results provide information on a second depletion and induction. No other studies have investigated the evolution of *A. filiformis* luminous capabilities later than one month after a single exogenous supply of coelenterazine. The depletion of the luminous capabilities following the first exogenous supply is faster than the depletion of the natural ones. The second induction period always presents lower values than the first, with slow increases. This phenomenon could be explained by the restricted amount of coelenterazine ingested, as a single boost, by the brittle star during the induction protocol compared to the amount available in the wild environment.

Moreover, the recorded wild-caught luminous capabilities suggest a nonrestricted amount of coelenterazine in the fjord, whereas a single boost of coelenterazine was given in captivity. Independently from the sampling season, quantifying the exact feeding rate and, by extension, the exact coelenterazine amount ingested by the brittle star during the induction remains challenging and could affect the reacquisition efficiency^24^. While Frank et al.,^17,18^ fed the shrimp *G. ingens* with luminous tissues containing coelenterazine without any cues on the luciferin concentration ingested, Haddock et al., 2001 overcame these issues while injecting a known amount of coelenterazine directly in *A. victoria* mesoglea^17,18^. Besides, *A. filiformis* arm regeneration status, age, gonad production, and other life history traits could explain the variability of luminous capabilities^24,25,27^.

In parallel with the luminometric assays, luciferase immunolabelling was conducted on the wild-caught brittle star. Delroisse et al.,^23^ immunodetected a specific *Renilla*-like luciferase in the inner part of the spine and the arm tip. The present immunolocalizations are consistent with these results^20,23^. This approach was used to pinpoint the photogenic cells within both tissues. No autofluorescent signal has been observed in these studies when paraformaldehyde-fixed samples were investigated^20,23^. Conversely, our study on unfixed arm tissues from wild-caught specimens highlights the presence of a bright green autofluorescent spot at the base of the spine. This autofluorescent signal co-localizes with the luciferase expression and with the specific cells identified by Delroisse et al.^23^ as the granular cell type II, the photogenic cells. The histology highlighted two distinctive spine types (sp I and sp II) co-occurring within a single arm segment. The current spine description is consistent with Delroisse et al.,^23^. The presence of similar cells in the inner tissues of spine types I and II was validated using light (on both paraffin and Spuur resin sections) as well as transmission electron microscopy methods. Those cells, characterized by their typical size, basal position, and appearance, correspond to the types I and II granular cells originally described ultrastructurally by Delroisse et al.,^23^. The luciferase immunolabelling and the ultrastructural description of the spine's inner cells led to the recognition of type II granular cells as the site of light production, the photocytes^23^. The paraffin sections highlight yellow-orange cells corresponding to the electron-dense cells found below the epidermal cell layer of the spine. These specific cells from the spine’s outer tissues match the type I granular cells identified as pigment cells^23^. According to our histological observations, the spine photogenic region is surrounded by a pigmentary sheath. Delroisse et al. hypothesized that the sheath facilitates light transmission from the photocyte to the tip of the spine^23^. A former study mentioned carotenoid pigments in *A. filiformis* tissue, essentially astaxanthin and lutein^41^. Moreover, carotenoids absorb blue light in the wavelength range of 400–500 nm^42,43^. The contribution of pigments associated with photocytes in enhancing light transmission has already been described in other luminous species, either through a melanin layer in elasmobranch (*e.g*., *Etmopterus spinax*, *Isistus brasilensis*)^44^ or a carotenoid layer in crustacean species (*e.g*., *Euphausia gibboides, Sergestes* species)^45,46^.

The coupling luminometric method and histological observations were needed to assess the autofluorescence signal's apparition and the luciferase's continuous expression within the arm spine and tip. Concomitant with the decrease of coelenterazine content during the depletion phase, the green autofluorescent signal disappears from the arm spine and tip. Conversely, during the induction phase, this signal reappears, consistently co-localized with luciferase within the photogenic cells at the base of the spine and the arm tip.

These histological observations could be linked with the luminometric recorded coelenterazine content before and after the food supply. Indeed, after coelenterazine intake, the amount of coelenterazine increases within the disk, followed by a rise in the arm tissue, suggesting a transfer of the luminous substrate from the stomach content to the luminous areas. The excitation and emission wavelength of the synthetic coelenterazine provided to the specimen matches the recorded wavelength. Therefore, the consistency between luminometric measurements and the histological observations lets us consider the recorded green autofluorescence as the coelenterazine signal for the first time.

Whether in the depletion or induction phase, luciferase activity and expression in *A. filiformis* tissue remain steady. Coubris et al.^39^ demonstrated that luciferase starts to be expressed after the juvenile settlement and metamorphosis from the planktonic larvae to the pentameric benthic individuals. Therefore, luciferase expression appears stable throughout the brittle star's benthic life.

To sum up, the European brittle star *A. filiformis* emits blue light via a coelenterazine-dependent luciferase, whose luminous capabilities evolve according to the coelenterazine exogenous acquisition throughout adult life. Luciferase is expressed throughout the benthic life; the only limiting factor for the bioluminescent reaction appears to be the coelenterazine available in the diet. No seasonal variation occurs, assuming a continuous presence of prey providing enough substrate to produce light. While the ultrastructure description of the spine tissue on fixed individuals is conserved, histology of unfixed specimens reveals green fluorescence dots attributed to the substrate presence, giving a new tool to spot both coelenterazine and photocytes. Work is underway to identify sources of coelenterazine in the brittle star’s diet and unveil the mechanism behind coelenterazine transfer from the stomach to the photogenic tissues.

### Supplementary Information

Supplementary Figure S1.

Supplementary Figure S2.

Supplementary Figure S3.

Supplementary Figure S4.

Supplementary Figure S5.

Supplementary Figure S6.

Supplementary Figure S7.

Supplementary Legends.

Supplementary Tables.

## Data Availability

Data is provided within the manuscript or supplementary information files.
