# Supplementary figures and images for: Maintain the light, long-term seasonal monitoring of luminous capabilities in the brittle star Amphiura filiformis
Source: Sci Rep. 2024 Jun 9;14:13238. doi: 10.1038/s41598-024-64010-x (PMC11163003; doi:10.1038/s41598-024-64010-x)

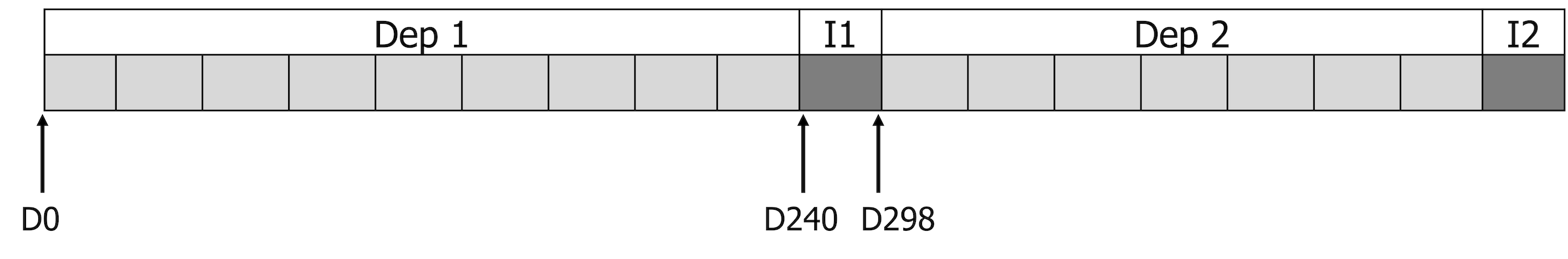

Supplement: Supplementary file 1 — Supplementary Figure S1. [file 41598_2024_64010_MOESM1_ESM.tiff]

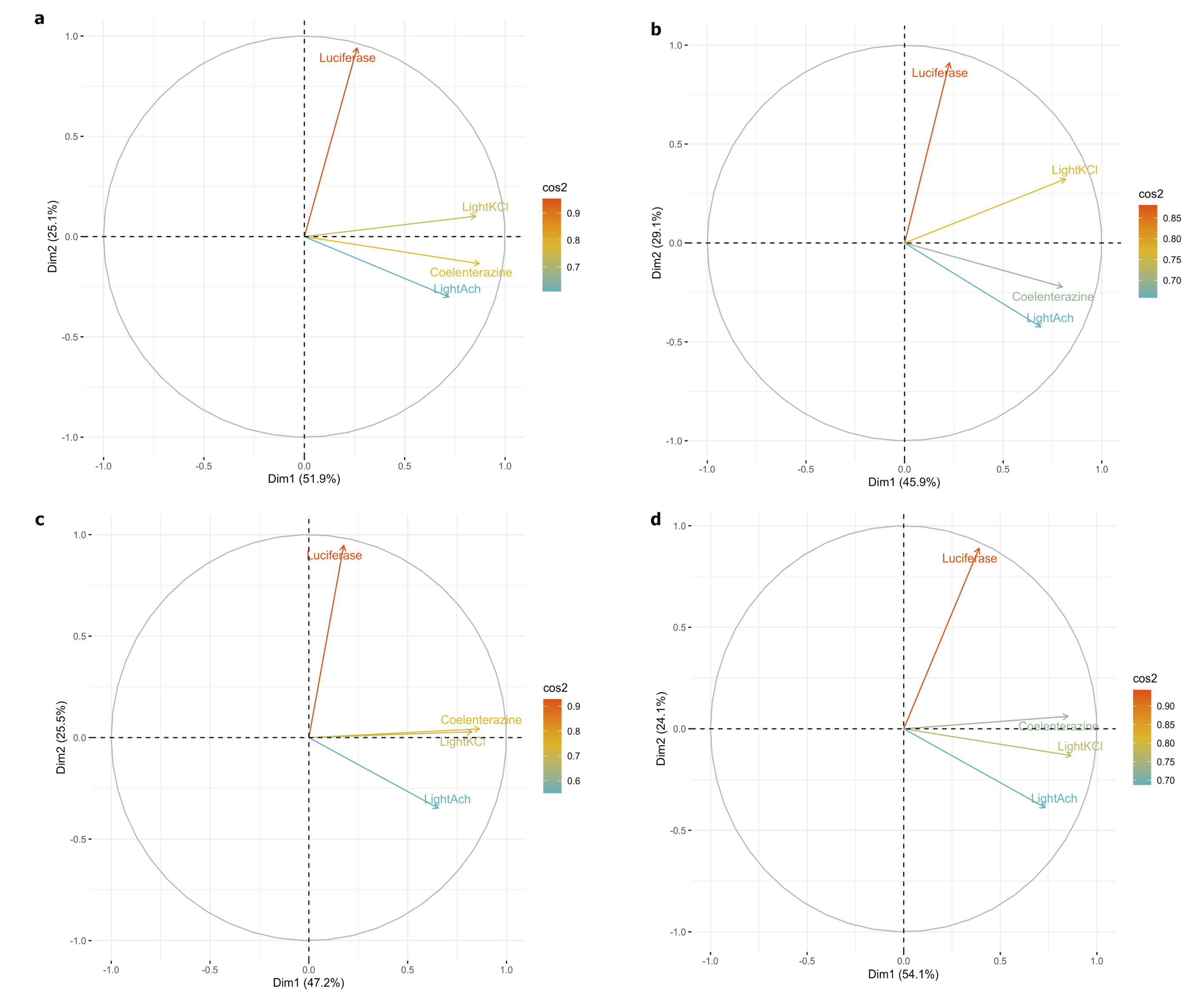

Supplement: Supplementary file 2 — Supplementary Figure S2. [file 41598_2024_64010_MOESM2_ESM.tiff]

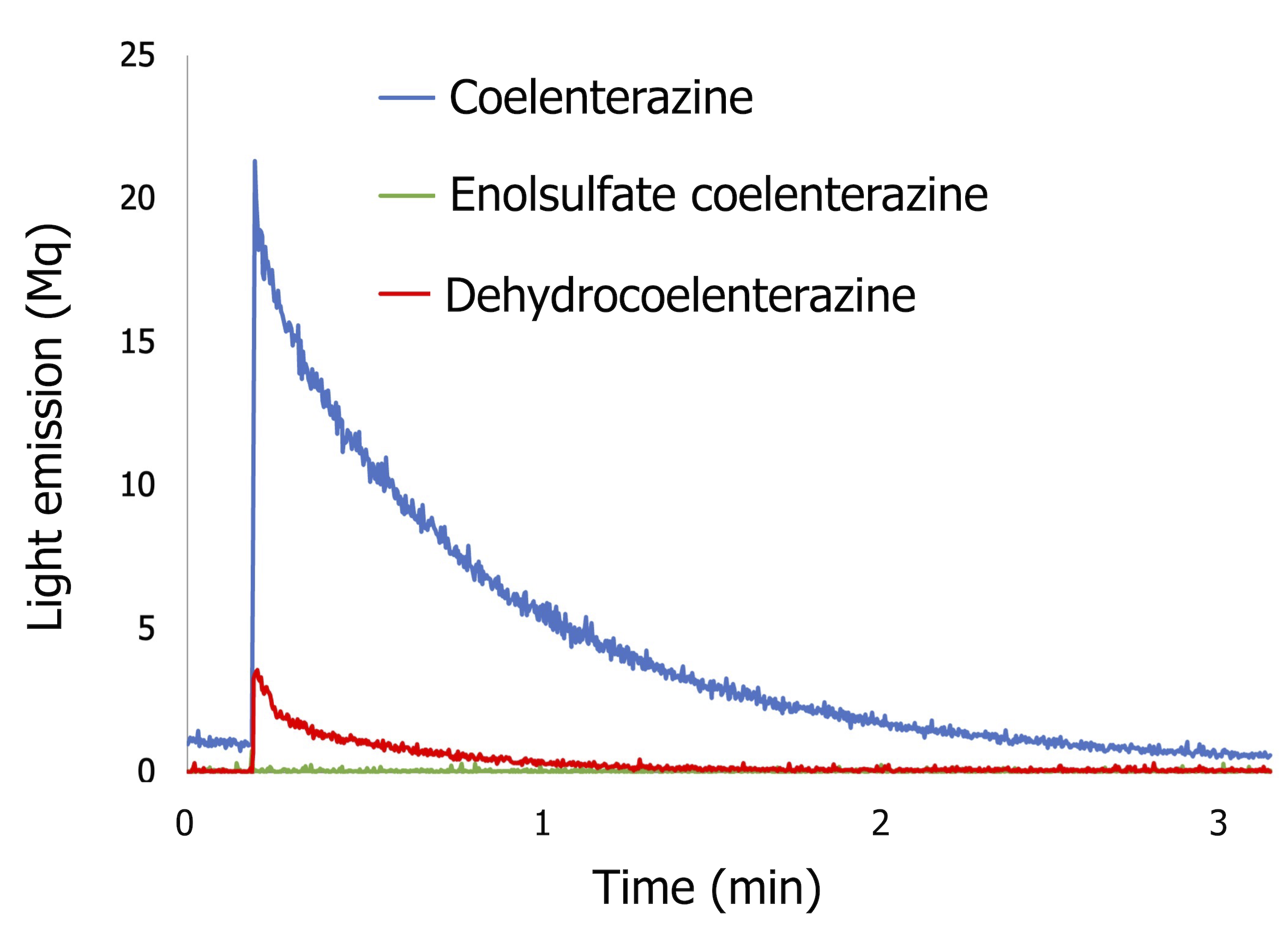

Supplement: Supplementary file 3 — Supplementary Figure S3. [file 41598_2024_64010_MOESM3_ESM.tiff]

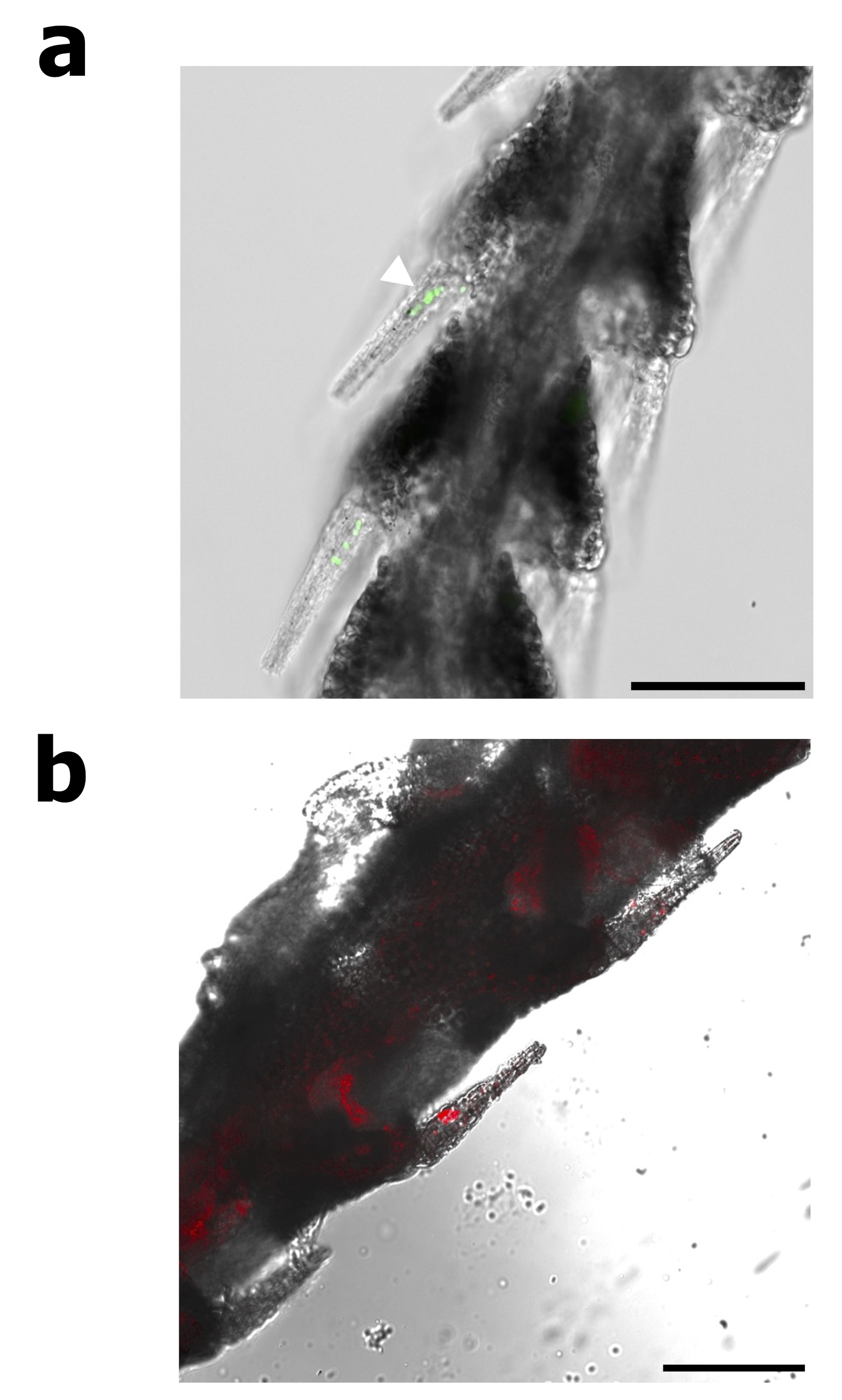

Supplement: Supplementary file 4 — Supplementary Figure S4. [file 41598_2024_64010_MOESM4_ESM.tiff]

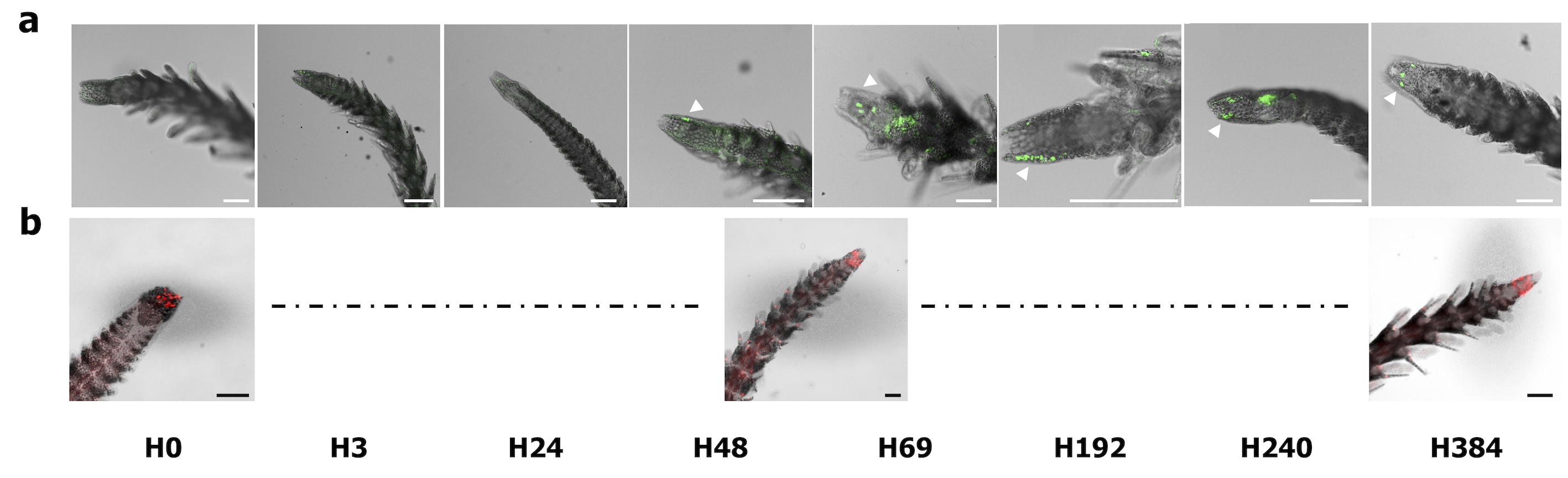

Supplement: Supplementary file 5 — Supplementary Figure S5. [file 41598_2024_64010_MOESM5_ESM.tiff]

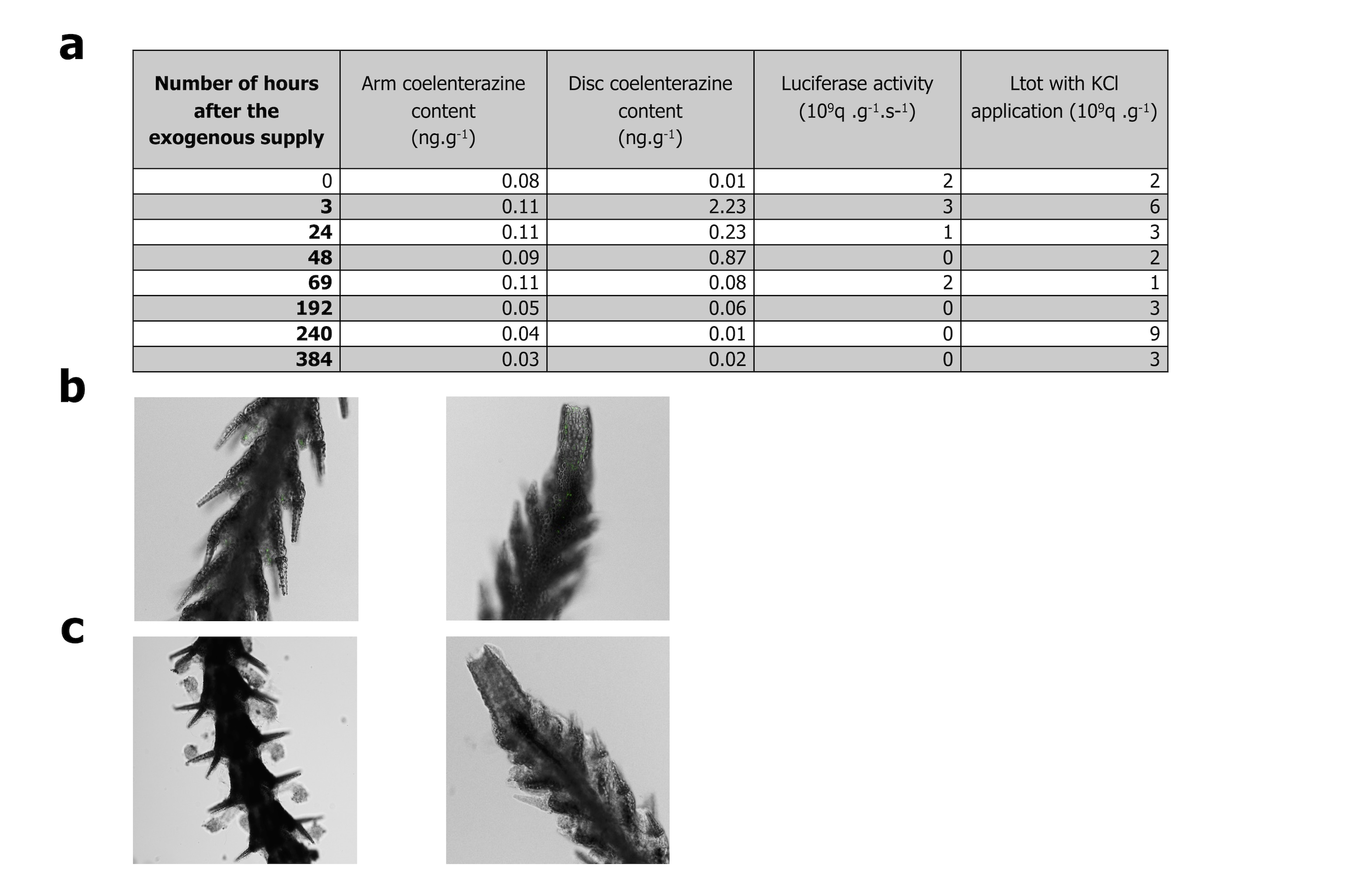

Supplement: Supplementary file 6 — Supplementary Figure S6. [file 41598_2024_64010_MOESM6_ESM.tiff]

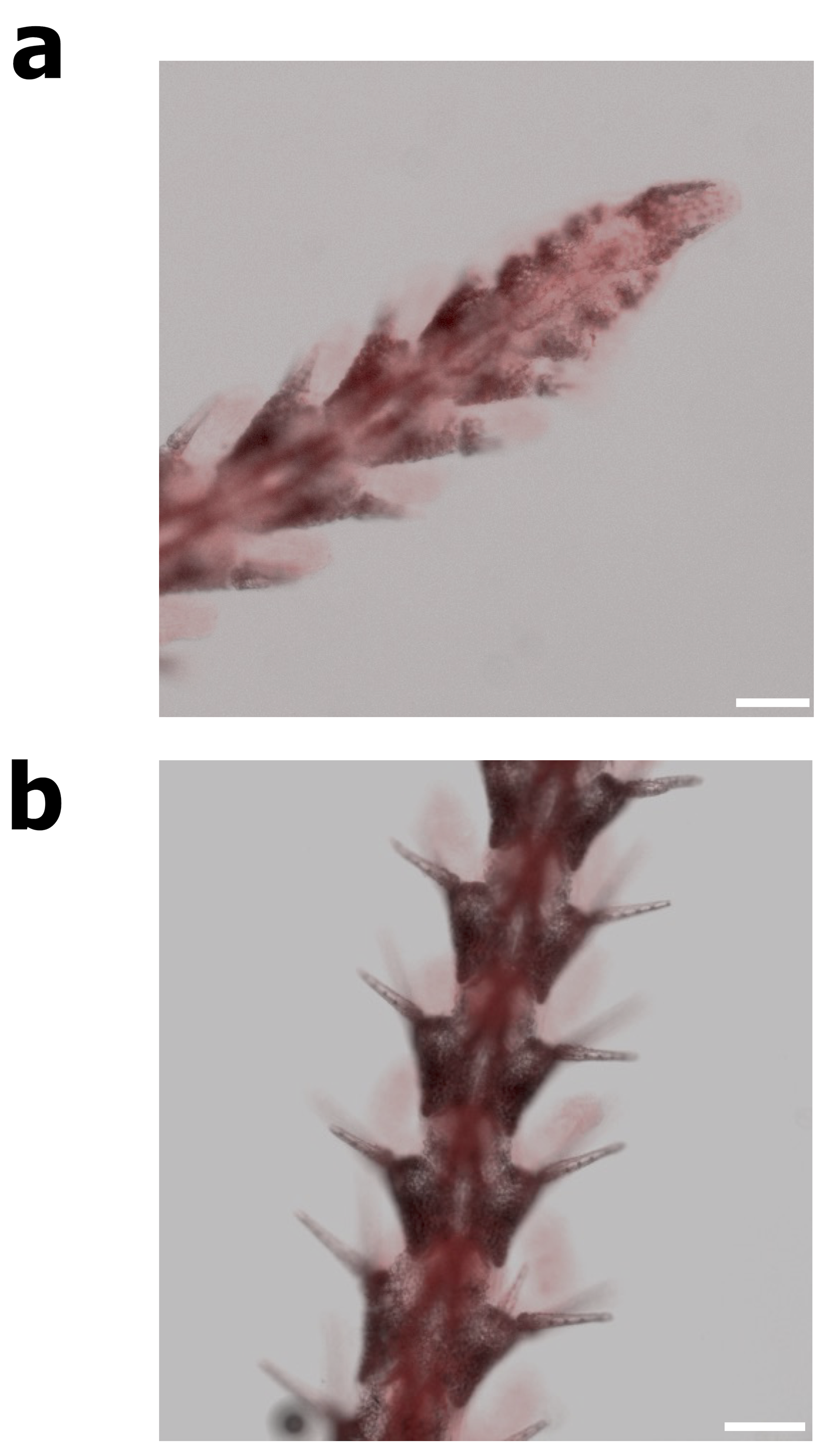

Supplement: Supplementary file 7 — Supplementary Figure S7. [file 41598_2024_64010_MOESM7_ESM.tiff]
