# Supplementary material for: Maintain the light, long-term seasonal monitoring of luminous capabilities in the brittle star Amphiura filiformis
Source: Sci Rep. 2024 Jun 9;14:13238. doi: 10.1038/s41598-024-64010-x (PMC11163003; doi:10.1038/s41598-024-64010-x)
Supplement: Supplementary file 8 — Supplementary Legends. [file 41598_2024_64010_MOESM8_ESM.docx]

**Supplementary information**

**Fig. S1: Experimental protocol for measuring luminous capabilities in *Amphiura filiformis* kept in captivity for a given season.** Each block corresponds to one measurement, monthly (light grey) or weekly (dark grey). Dep 1: First depletion period; I1: First induction period; Dep 2: Second depletion period; I2: Second induction period. For the winter season, Dep 2 is restricted to six months.

**Fig. S2: Principal Component Analysis performed on the recorded luminometric parameters (coelenterazine content, luciferase activity, and total light emission values under KCl and Ach applications) for (a) summer 2021, (b) fall 2021, (c) winter 2022, and (d) spring 2022.**

**Fig. S3: Typical curves obtained during the coelenterazine storage forms assays, enolsulfate coelenterazine, and dehydrocoelenterazine.**

**Fig. S4: Histological analysis of wild-caught *A. filiformis*.** Natural autofluorescence signal (green; white arrowhead) and immunodetection (red) of luciferase expression within the arm’s spine.

**Fig. S5: Monitoring the luminometric parameters, coelenterazine autofluorescence, and luciferase immunodetection in the arm’s tips before and after induction.** (a) Luminometric parameters were recorded after a single boost of coelenterazine over 384 hours. Green bars correspond to the coelenterazine content (ng g^−1^) in arms tissue, dark green bars correspond to the coelenterazine content (ng g^−1^) in the disk, red bars correspond to the luciferase activity (10^9^ q g^−1^ s^−1^), and black bars correspond to the Ltot with KCl application (10^9^ q g^−1^). Values are expressed as mean ± s.e.m. Asterisks indicate statistical differences between measurements performed before (H0) and after a boost of coelenterazine (n = 6). According to the parametric assumptions, either one-way ANOVA and Dunnett’s multiple comparisons test or Kruskal–Wallis ANOVA and Dunn multiple comparisons test comparisons test were performed. Statistical differences were highlighted with *P-value<0.05. (b) Following the autofluorescence signal (green; white arrowhead) within the arm’s tips before and after an exogenous supply of coelenterazine. (c) Immunodetection (red) of luciferase expression within the arm’s tips before induction and at 50 and 384 hours after an exogenous supply of coelenterazine. Scale bar = 100 µm.

**Fig. S6: Luminometric, histological, and immunohistological control performed on the non-luminous brittle star *A. chiajei*.** (a) Luminometric parameters (coelenterazine content, luciferase activity, and Ltot after KCl application) were recorded after a single boost of coelenterazine over 384 hours. (b) Autofluorescence signal (green) (c) and immunodetection (red) (bottom) of luciferase expression within the arm’s spine and tips after an exogenous supply of coelenterazine. Scale bar = 100 µm.

**Fig. S7: Immunodetection controls without the primary antibody in *A. filiformis***. Scale bar = 100 µm.

**Table S1: Luminometric values and statistics for wild-caught *Amphiura filiformis* individuals.** Mean values and the s.e.m of the coelenterazine content (ng g^−1^) in arms tissue, the luciferase activity (10^9^ q g^−1^ s^−1^), the Ltot with KCl application (10^9^ q g^−1^), and the Ltot with Ach application (10^9^ q g^−1^) statistical test and alpha level significativity for each sampling seasons (n = 30).

**Table S2: Luminometric values and statistics obtained during the seasonal, long-term monitoring of *Amphiura filiformis*.** Mean values and the s.e.m of the coelenterazine content (ng g^−1^) in arms tissue, the luciferase activity (10^9^ q g^−1^ s^−1^), the Ltot with KCl application (10^9^ q g^−1^), and the Ltot with Ach application (10^9^ q g^−1^) including sample size, statistical test and alpha level significativity for each luminometric measurements of the sampling seasons.

**Table S3: Luminometric values and statistics obtained before and after an induction performed on *Amphiura filiformis*.** Mean values and the s.e.m of the coelenterazine content (ng g^−1^) in arms tissue, the coelenterazine content (ng g^−1^) in the disk, the luciferase activity (10^9^ q g^−1^ s^−1^), and the Ltot with KCl application (10^9^ q g^−1^) including sample size, statistical test and alpha level significativity after a single boost of coelenterazine over 384 hours.
